# *Rhipicephalus microplus* and *Ixodes ovatus* cystatins in tick blood digestion and evasion of host immune response

**DOI:** 10.1186/s13071-015-0743-3

**Published:** 2015-02-24

**Authors:** Luís Fernando Parizi, Gabriela Alves Sabadin, María Fernanda Alzugaray, Adriana Seixas, Carlos Logullo, Satoru Konnai, Kazuhiko Ohashi, Aoi Masuda, Itabajara da Silva Vaz Jr

**Affiliations:** Centro de Biotecnologia, Universidade Federal do Rio Grande do Sul, Avenida Bento Gonçalves, 9500, Prédio 43421, Porto Alegre, 91501-970, RS Brazil; Department of Disease Control, Laboratory of Infectious Diseases, Graduate School of Veterinary Medicine, Hokkaido University, 060-0818 Sapporo, Hokkaido Japan; Departamento de Ciencias Microbiológicas, Laboratorio de Inmunología, Facultad de Veterinaria, UDELAR, Montevideo, Uruguay; Departamento de Farmacociências, Universidade Federal de Ciências da Saúde de Porto Alegre, Rua Sarmento Leite, 245, Porto Alegre, 90050-170, RS Brazil; Laboratório de Química e Função de Proteínas e Peptídeos–CBB–UENF and Unidade de Experimentação Animal, Avenida Alberto Lamego, 2000, Horto, Campos dos Goytacazes, 28015-620, RJ Brazil; Faculdade de Veterinária, Universidade Federal do Rio Grande do Sul, Avenida Bento Gonçalves, 9090, Porto Alegre, 91540-000, RS Brazil; Instituto Nacional de Ciência e Tecnologia em Entomologia Molecular, Rio de Janeiro, Brazil

**Keywords:** Inhibitor, Cystatin, Tick, *Rhipicephalus microplus*, *Ixodes ovatus*

## Abstract

**Background:**

Cystatins are a group of cysteine protease inhibitors responsible for physiological proteolysis regulation and present in a wide range of organisms. Studies about this class of inhibitors in parasites have contributed to clarify their roles in important physiological processes, like blood digestion and modulation of host immune response during blood feeding. Thus, cystatins are a subject of research on the development of new parasite control methods. Additionally, the characterization of proteins shared by different parasite species represents a valuable strategy to find potential targets in multi-species control methods. However, cystatin functions in ticks remain undetermined, especially in *Rhipicephalus microplus* and *Ixodes ovatus*, two species that affect livestock and human health, respectively.

**Methods:**

Here we report the inhibitory profile of two *R. microplus* (BrBmcys2b and BrBmcys2c) and one *I. ovatus* (JpIocys2a) cystatins to commercial cathepsins B, C, and L. The presence of native cystatins in *R. microplus* tissues was analyzed using sera against recombinant BrBmcys2b and BrBmcys2c. Also, a peptide from JpIocys2a was synthesized for rabbit immunization, and this serum was used to analyze the cross antigenicity between *R. microplus* and *I. ovatus* cystatins.

**Results:**

Enzymatic inhibition profile of tick cystatins shows a distinct modulation for cathepsins related to tick blood digestion and evasion of host immune response. Furthermore, BrBmcys2b was detected in saliva and different tissues along tick stages, while BrBmcys2c was detected mainly in gut from partially engorged *R. microplus* females, demonstrating a distinct pattern of cystatin expression, secretion and traffic between tick tissues. Moreover, phylogenetic analysis suggests that JpIocys2a belongs to the group of tick gut secreted cystatins. Finally, cross-antigenicity assays revealed that antibodies against the JpIocys2a peptide recognize native and recombinant *R. microplus* cystatins.

**Conclusion:**

The presence of these proteins in different tissues and their ability to differentially inhibit cathepsins suggest distinct roles for JpIocys2a, BrBmcys2b, and BrBmcys2c in blood digestion, egg and larvae development, and modulation of host immune response in tick physiology. The cross-antigenicity between native and recombinant cystatins supports further experiments using JpIocys2a, BrBmcys2b, and BrBmcys2c as vaccine antigens.

## Background

Cystatins are tightly binding inhibitors of cysteine proteases [1]. In parasites, these inhibitors are involved in internal protective and regulatory biological processes [2] as well as in the modulation of the host’s defense responses [3]. Classically, cystatins are divided into three groups, known as stefin (type 1), cystatin (type 2), and kininogen (type 3), although in ticks only stefins and cystatins have been reported [4]. It was only in recent years that the physiological functions of tick cystatins [4,5] and their target cysteine cathepsins [6,7] during tick blood-feeding, digestion and development became the object of more consistent research. Previous studies have also demonstrated that tick cystatins modulate host cathepsins involved in processes like inflammation, antigen processing and presentation, phagocytosis, and cytokine expression [2]. The regulation of these physiological processes by tick cystatins promotes blood uptake and survival of parasite while it is attached to the host.

The first tick cystatin biochemically or molecularly characterized was isolated from *Amblyomma americanum* [8]. Back then, the participation of this cystatin was implicated in host immunomodulation and tick protection to harmful ingested factors during blood feeding. In *Ixodes scapularis*, two cystatins were biochemically characterized, Sialostatin L and Sialostatin L2 [9,10]. Sialostatin L showed host immune system modulation and cathepsins L, V, C, X, B, and papain inhibition, while Sialostatin L2 inhibited cathepsins L, V, S, and C, showing higher expression rate in late feeding. The enzymatic inhibition profile of *Haemaphysalis longicornis* cystatins for papain, cathepsins L, B, H, as well as tick cathepsins have been characterized in previous research [7,11-14]. Furthermore, it was demonstrated that some of the cystatins from *H. longicornis* play a role in innate immunity [11] and blood feeding [7,14]. Cystatins from *Ornithodoros moubata* were able to inhibit cathepsins B, L, S, H, and C [15,16], and affect T-cell and dendritic cells proliferation and cytokine release [16]. Additionally, it was suggested that one *Rhipicephalus appendiculatus* cystatin present in nymph, male and female gut after feeding is involved in blood digestion process [17]. Taken together, these results indicate that cystatins play widespread and distinct regulatory roles in different tick species.

*Rhipicephalus microplus* is one of the most consistently studied cattle tick species, mainly because of the potentially expressive economic losses it causes in the livestock industry [18]. However, despite the great interest in understanding the physiology of this tick, few studies have analyzed *R. microplus* cystatins. Some *R. microplus* cysteine proteases were identified and characterized [19-21], demonstrating the importance of these enzymes in a variety of physiological processes and parasite stages. Nevertheless, few *R. microplus* cystatins and its target cysteine proteases have been characterized, including only one type 2 cystatin [5], named Rmcystatin-3. Rmcystatin-3 is expressed in tick fat body, salivary glands, and hemocyte, though it inhibits cathepsin L, B, and BmCl-1, a gut *R. microplus* cysteine endopeptidase [19], which suggests its role in tick blood digestion. Consequently, the control of *R. microplus* cysteine proteases activities by cystatins remains essentially unknown. In a previous work [22] we analyzed the sequence properties and immunogenicity of putative cystatins from *R. microplus*. These cystatins showed a high degree of homology among *Rhipicephalus* spp., differential RNA expression patterns in tick tissues, as well as cross-reactivity between them, suggesting the existence of shared epitopes.

The tick *Ixodes ovatus* geographic prevalence has been reported in Southeast Asia countries [23,24]. Its main hosts are humans, and bite cases have been observed in Tibet, Burma, Nepal, Japan, and China [25]. *Borrelia burgdorferi* and *Ehrlichia* species, which are the causal agents of Lyme disease and ehrlichiosis, respectively, are transmitted by ixodid ticks, and *I. ovatus* was found to be infected with *Borrelia* and *Ehrlichia* species [26-28]. However, no human cases of Lyme disease and ehrlichiosis transmitted by *I. ovatus* have been confirmed to date [27]. Also, no cystatins were characterized for this tick species so far.

Tick control is a great challenge in livestock and public health management worldwide, and relies on the use of synthetic acaricides [29]. In spite of that, vaccines have emerged as an interesting alternative method to decrease tick populations and the incidence of tick-borne diseases in the environment [30]. Since hosts in several regions are exposed to multi-tick infestation, the development of a single vaccine against multiple species may be advantageous in control strategies against these parasites. A number of vaccination experiments showed the potential use of tick proteins to protect hosts against more than one tick species, demonstrating the feasibility of induction of cross-protection [31]. These tick protective proteins are present in many physiological processes, like Bm86, a gut protein of unknown function [32]; glutathione-S transferase, an enzyme responsible to detoxification of cell xenobiotic compounds [33]; ferritins, iron-storage proteins [34]; a cement protein named 64TRP [35]; and subolesin, a gene-expression regulator [36]. In fact, some of these tick antigens, such as Bm86 and its homologues, developed a higher protective host immune response to different tick species, rather than the tick species from which the antigen was isolated [31]. Vaccination trials using cystatins as antigens were performed against *I. scapularis* [37] and *Ornithodoros moubata* [16] infestations, showing the potential of these inhibitors to compose a vaccine against ticks. Despite the importance of cross antigenicity analysis as a preliminary step to detect multi-species antigen candidates, the potential of cross-protection induced by tick cystatins has yet to be analyzed.

In order to improve the understanding of the physiological roles of cystatins and the potential of these inhibitors as antigen in a multi-species vaccine, we characterized the inhibitory profile, tissue expression, and cross-antigenicity of a new *I. ovatus* cystatin, JpIocys2a, and two *R. microplus* cystatins, BrBmcys2b and BrBmcys2c. The presence of these proteins in different tick tissues and their ability to differently inhibit cathepsins suggest distinct roles for JpIocys2a, BrBmcys2b, and BrBmcys2c in blood digestion and modulation of host immune response in tick physiology. The cross-antigenicity among cystatins from these two tick species paves the way for further experiments using JpIocys2a, BrBmcys2b, and BrBmcys2c as vaccine antigens.

## Methods

### Animals and ticks

Partially and fully engorged female ticks (Porto Alegre *R. microplus* strain) were collected from Hereford (*Bos taurus taurus*) cattle for tissue dissection. Partially engorged *R. microplus* females weighing between 25 and 60 mg were recovered manually from calves [38]. New Zealand White rabbits and Hereford cattle were housed at the Faculdade de Veterinária of the Universidade Federal do Rio Grande do Sul, Brazil. The experiments were approved and conducted following the guidelines of the Ethics Committee on Animal Experimentation of the same university. *I. ovatus* adult ticks were collected by flagging from the lower vegetation in forests in Hokkaido, Japan, and maintained by experimental infestation on hamsters to full engorgement. Hamsters were maintained in a P3 animal facility at Graduate School of Veterinary Medicine, Hokkaido University in accordance with the Institutional Animal Care and Use Committee guidelines.

### Cloning of cystatin ORF sequences

For cloning of the *JpIocys2a* sequence, primers 5′-GACTAGTCGCCAGCACGATGGCT-3′ (forward) and 5′-TGTCATTTAACATGCGGCTGACGTC-3′ (reverse) were designed based on TIGR nucleotide database (TC51659) from *I. scapularis* gene sequence with high similarity to cystatins. One ORF was amplified by RT-PCR from *I. ovatus* ovary RNA and cloned in pGEM-T vector (Promega). For cloning the DNA sequence encoding the mature JpIocys2a protein in the expression vector, the plasmid pGEM-T-JpIocys2a was amplified using the primers 5′-TTTTTGGATCCGGGTCGGCGAGCAGGTC-3′ (forward) and 5′-AAAAAGAATTCCTAGACATTATTAGGAGCTTCGCAGTGGTAG-3′ (reverse). The PCR product was hydrolyzed with *Bam* HI and *Eco* RI (Invitrogen) restriction enzymes and separated by electrophoresis on 0.8% agarose gel. The 396-bp fragment was excised, purified using the Geneclean II Kit (Qbiogene), and ligated into plasmid pGEX-4 T-1 (GE Healthcare) downstream the Glutathione S-transferase (GST) gene, which codifies the fusion protein. The plasmid pGEX-4T-1-JpIocys2a was transformed into *Escherichia coli* XL1BLUE strain. Transformed cells were plated and cultivated in LB agar containing ampicillin and chloramphenicol (50 μg/mL). The plasmid was purified and the cloned product was confirmed by sequencing.

The BrBmcys2b and BrBmcys2c nucleotide sequences were previously cloned [22]. In short, cystatin-coding regions were amplified by PCR from *R. microplus* salivary gland cDNA and cloned into plasmid pET-5a (Novagen).

### *In silico* analyses

JpIocys2a DNA sequence analysis, amino acid predictions, and sequences alignment were conducted using the BioEdit version 7.2.5 software [39]. The cystatin antigenic index was calculated using the Jameson–Wolf algorithm in the software LASERGENE, version 7.0.0, to predict antigenic determinants by combining existing methods for protein structural predictions [40]. For phylogenetic analysis, an unrooted neighbor-joining phylogenetic tree was created using the MEGA software, version 5 [41]. Bootstrap support was assessed using 1000 replicates. The GenBank accession numbers for cystatins used in analysis were: *R. microplus* BrBmcys2b [GenBank: KC816580], *R. microplus* BrBmcys2c [GenBank: KC816581], *R. microplus* Rmcystatin-3 [GenBank: AIX97454]; *I. ovatus* JpIocys2a [GenBank: KP253747]; *I. scapularis* sialostatin 1 [GenBank: AF483724], *I. scapularis* sialostatin 2 [GenBank: DQ066048]; *H. longicornis* Hlcyst-2 [GenBank: DQ364159], *H. longicornis* Hlcyst-3 [GenBank: EU426545].

### Peptide synthesis

To raise antibodies against conserved and exposed regions among tick cystatins, a peptide with 14 amino acids from the JpIocys2a amino acid sequence was synthesized (STQpep). Antigenic analysis of cystatins and percentage of identity among JpIocys2a, BrBmcys2a, BrBmcys2b, Brbmcys2c, and other tick cystatins were used to select the peptide. Peptides were kindly provided by Maria Aparecida Juliano, Department of Biophysics, Federal University of São Paulo (UNIFESP), SP, Brazil. An automated bench-top simultaneous multiple solid-phase peptide synthesizer (PSSM 8 system, Shimadzu) was used for the solid-phase synthesis of the peptides by the Fmoc-procedure. Final peptides were deprotected in trifluoracetic acid and purified by semipreparative HPLC using an Econosil C-18 column. Analytical HPLC was performed using a binary HPLC system from Shimadzu with a SPD-10AV Shimadzu UV–vis detector, coupled to an Ultrasphere C-18 column. The HPLC column elutes were monitored by their absorbance at 220 nm. The molecular weight and purity of synthesized peptides were checked by MALDI-TOF mass spectrometry (Bruker Daltons) or electron spray LC/MS-2010 (Shimadzu) [42,43].

### Expression and purification of recombinant cystatins

*E. coli* C41 (DE3), C43 (DE3) and RIL strains were transformed with plasmids containing the BrBmcys2b, BrBmcys2c, and JpIocys2a sequences, respectively. The recombinant proteins BrBmcys2b and BrBmcys2c were expressed in SOB medium with 0.4 mM isopropyl-β-D-thiogalactopyrano (IPTG) for 16 h at 25°C. rGST-JpIocys2a protein was expressed in LB medium with 0.1 mM IPTG for 24 h at 37°C. Cells were further harvested by centrifugation at 10,000 × g for 10 min at 4°C. rBrBmcys2b and rBrBmcys2c were resuspended in phosphate buffer containing 100 mM of imidazole (lysis buffer) and rGST-JpIocys2a in PBS. For cell lysis, the suspension was sonicated five times for 30 s at 40 MHz on ice (Sonics or Qsonica, respectively). The soluble and insoluble fractions were separated by centrifugation at 10,000 × g for 10 min at 4°C (Hitachi).

The soluble fractions containing the rBrBmcys2b and rBrBmcys2c were purified by nickel-chelating Sepharose chromatography (GE Healthcare). Briefly, the soluble fractions were filtered in 0.45-μm filters (Whatman) and then applied into the columns previously equilibrated with phosphate lysis buffer. Proteins of interest were eluted with phosphate buffer containing 150 mM of imidazole at room temperature. Eluted fractions were purified using centrifugal filter devices (Centricon YM10 - 50,000 MW cut-off, Millipore), lyophilized and dialyzed against PBS. The soluble fractions containing the rGST-JpIocys2a was purified by Glutathione Sepharose 4B (Amersham Bioscience) and eluted with 50 mM Tris–HCl buffer and 10 mM glutathione, in pH 8.0. Purified rGST-JpIocys2a was dialyzed in cleavage buffer (NaCl 140 mM, KCl 2.7 mM, Na_2_HPO_4_ 10 mM, KH_2_PO_4_ 1.8 mM, pH 7.3) and cleaved by Thrombin (0.01u/μg; Sigma). rGST fusion protein was bound by Glutathione Sepharose 4B, and pure rJpIocys2a was recovered in the supernatant. Protein concentrations were determined using the BCA Protein Assay kit (Thermo Scientific) following the manufacturer’s instructions.

### Enzymatic assays

Remaining enzymatic activity and apparent dissociation constants (KIs) were estimated using different cathepsins (Sigma) to determine the inhibitory profile of rBrBmcys2b, rBrBmcys2c, and rJpIocys2a. Enzymes were preincubated with recombinant cystatins at different concentrations ranging from 10 to 500 nM in the corresponding assay buffer for 15 min, and protease-specific substrates (Sigma) were added to estimate residual enzyme activity. Enzymes concentrations are presented in Table 1. Assay buffer and enzymes were used as follows: 100 mM sodium acetate, pH 5.5, 100 mM NaCl, 1 mM EDTA, and 0.005% TritonX-100 for bovine cathepsin C and human cathepsin L; 100 mM sodium acetate, pH 5.5, 60 mM NaCl, 1 mM EDTA for bovine cathepsin B; 100 mM HEPES, pH 7.5, 1 mM EDTA for human cathepsin G. For KIs determination, substrates were used as follows: Z-Phe-Arg-MCA (0.012-0.1 mM) for cathepsin L; Z-Arg-Arg-pNA (0.012-1.0 mM) for cathepsin B; Gly-Phe-pNA (0.6-2.25 mM) for cathepsin C; N-Succinyl-Ala-Ala-Pro-Phe-pNA (0.67 mM) for cathepsin G. Chromogenic (405 nm of absorption) and fluorescence intensity (370 and 460 nm for emission and excitation, respectively) assays were monitored in a microplate spectrophotometer (Spectramax Microplate Reader, Molecular Devices Corporation). Data were fit for appropriate tight-binding inhibitors using a nonlinear regression analysis equation (Morrison, 1969) performed using GraphPad Prism version 5.00 for Windows (GraphPad Software).

**Table 1:** **Cystatins dissociation constants (KIs) for different proteases**

**Enzyme**	**Family**	**Enzyme concentration**	**Ki (nM)**		
			**rBrBmcys2b**	**rBrBmcys2c**	**rJpIocys2a**
Cathepsin B ^1^	Cysteine protease	0.500 μM	0.82 ± 0.35	n.i.	154.70 ± 106.71
Cathepsin C ^1^	Cysteine protease	0.160 μM	26.65 ± 6.75	0.45 ± 0.12	>1 μM
Cathepsin L ^2^	Cysteine protease	0.043 μM	2.48 ± 1.00	28.45 ± 2.34	4.39 ± 1.76
Cathepsin G ^2^	Serine protease	0.100 μM	n.i	n.i.	n.i.

n.i., not inhibited in the presence of 0.5 μM recombinant cystatin.

^1^Bovine cathepsin.

^2^Human cathepsin.

### Tick tissues extraction

Salivary glands, ovary, gut, fat body and larvae were disrupted and homogenized using a mortar and pestle in an ice bath with 10 mM phosphate buffer, pH 7.2. The homogenate was centrifuged at 16,000 × g for 15 min at 4°C to remove the insoluble material. Next, the soluble supernatant fraction was collected. The protein extracts were prepared according to the method previously described [44]. Tick hemolymph was collected as follows: engorged *R. microplus* females were washed in alcohol 70%, fixed to Petri dishes and kept at 4°C; the cuticle was slit with a razor blade and the exuding hemolymph recovered and stored at −20°C. Saliva was collected by pilocarpine injection as previously described [45].

### Immunization of hamsters and rabbits

Two hamsters were subcutaneously inoculated with 50 μg of rBrBmcys2c or PBS. Immunizations consisted of four doses at 14-day intervals with the recombinant proteins emulsified in Freund’s incomplete adjuvant. Two rabbits were subcutaneously immunized four times at 14-day intervals with 625 μg of STQpep conjugated with carrier protein keyhole limpet hemocyanin (KLH; Sigma) or 200 μg of GST from *H. longicornis* [46] both emulsified in Marcol-Moltanide (Exxon Mobil Corporation) adjuvant. Hamsters and rabbit blood were collected 14 days after the last booster, and sera were separated by centrifuging samples at 10,000 × g for 5 min at 4°C. Serum aliquots were preserved at −20°C upon use. Mice sera used in serological analysis were produced in a previous study [22].

### SDS-PAGE and Western blotting

The production of recombinant cystatins and the presence of native cystatin in tissues were analyzed by SDS–PAGE [47] and Western blot [48]. For Western blot analyses, recombinant cystatins (1 μg protein/lane), saliva (20 μg protein/lane), hemolymph (60 μg protein/lane) and tissue extracts (150 μg protein/lane) were resolved in 14% gel for SDS-PAGE, followed by transference to nitrocellulose membranes. The membranes were blocked with 5% non-fat dry milk in PBS and further incubated with mice, hamster or rabbit sera diluted to 1:50. Additionally, for rBmcys2b, rBmcys2c, and rJpIocys2a detection, anti-histidine tag or anti-GST-Hl antibodies (1:2,000) were used. After primarily sera incubations, anti-IgG species specific alkaline phosphatase (mouse and rabbit sera) and peroxidase (hamster serum) conjugates (1:5,000) were used as secondary antibodies. Alkaline phosphatase revelations were performed with NBT (nitro blue tetrazolium) and BCIP (5-bromo-4-chloro-3-indolyl phosphate, Sigma) in PBS. Peroxidase revelations were performed with DAB (3,3′-diaminobenzidine tetrahydrochloride), H_2_O_2_ and CoCl_2_ in PBS.

## Results

### JpIocys2a identification and sequence analyses

**Figure 1:**
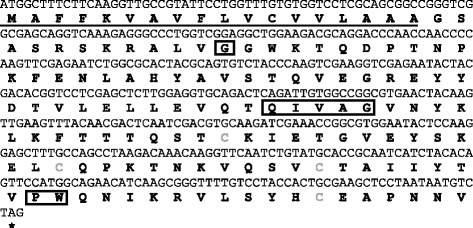
**JpIocys2a nucleotide and predicted amino acid sequences.** The predicted signal peptide (SignalP) is underlined. Cysteine residues are in gray and the conserved cystatin motifs PI (G), P II (QxVxG), and P III (PW) are boxed.

JpIocys2a nucleotide sequence showed an ORF of 423 bp, and the deduced amino acid sequence contained a signal peptide with cleavage site between amino acid residues 18 and 19 and four cysteine residues with a theoretical molecular weight of 15.6 kDa (Figure 1). The G residue on N-terminal, the motif QxVxG and the PW at the C-terminal are characteristic of family 2 cystatins, and are also conserved in JpIocys2a. The SND inhibitory domain, which inhibits legumain/asparaginyl endopeptidases found in human cystatin C, is absent in JpIocys2a, like other tick cystatins [4].

### *In silico* antigenicity of cystatins and peptide selection

**Figure 2:**
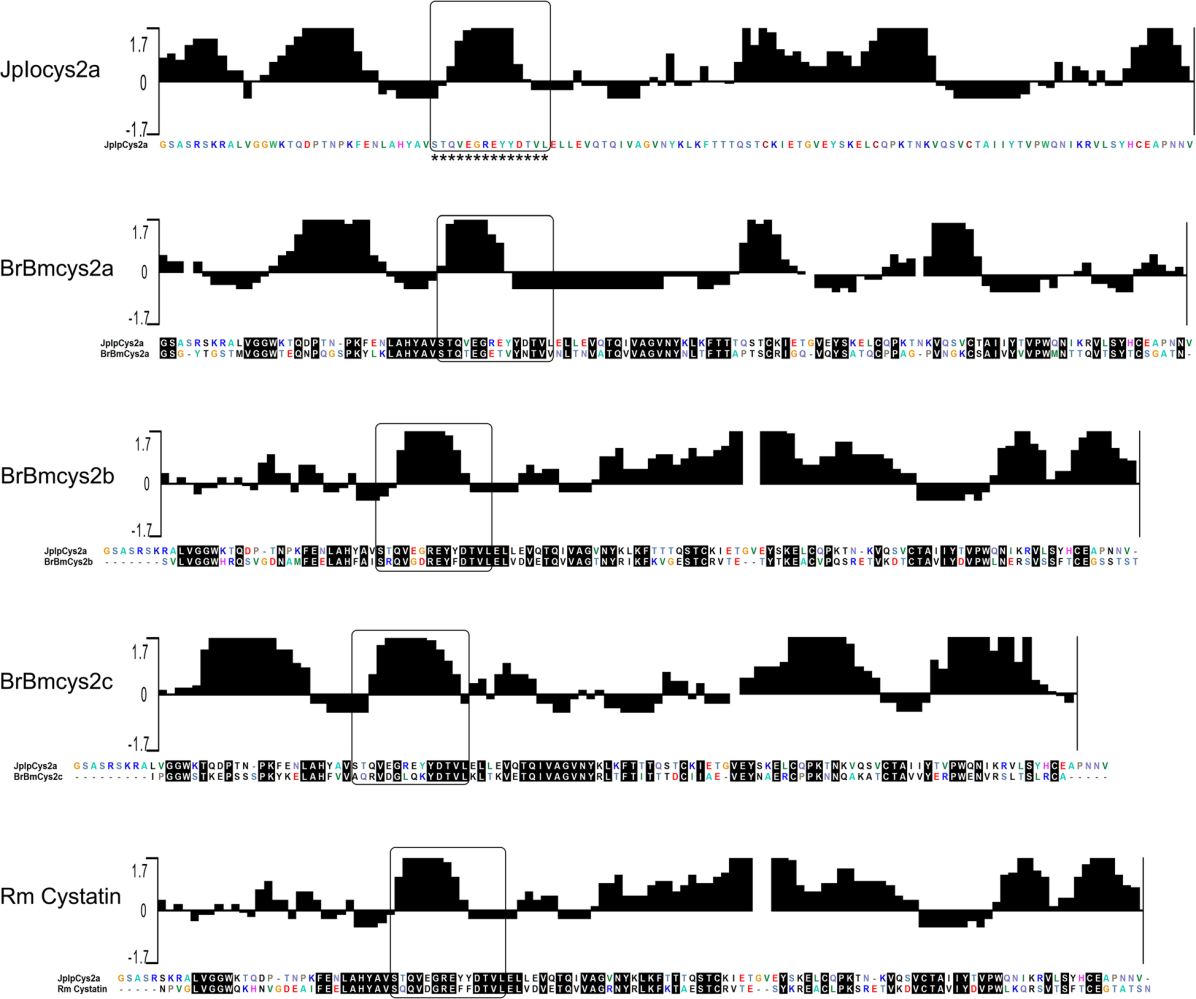
**Conserved and antigenic tick cystatin regions for JpIocys2a peptide selection.** Antigenic index plots for tick cystatins were predicted using the Jameson–Wolf algorithm. Graphic increased positivity shows predictive antigenic sites. Alignment shows conserved regions between JpIocys2a and *R. microplus* cystatins. Black boxes indicate conserved and antigenic amino acid region for each sequence. Asterisk indicate the selected region for peptide synthesis.

The alignment of JpIocys2a with *R. microplus* cystatins showed conserved regions among the predicted amino acid sequences (Figure 2). The amino acid sequence STQVEGREYYDTVL from JpIocys2a (STQpep) was selected for peptide synthesis in accordance with the highest identity and antigenic region among all cystatins analyzed.

### Production of recombinant cystatins

**Figure 3:**
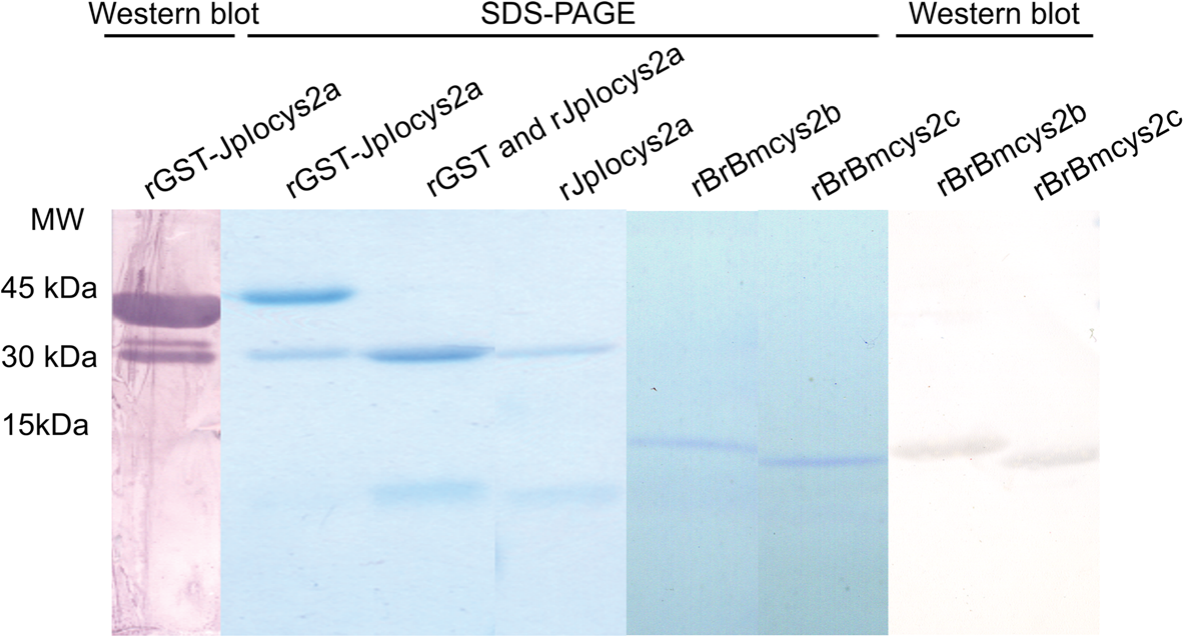
**SDS-PAGE and Western blot of rJpIocys2a, rBrBmcys2b and rBrBmcys2c production.** Western blot: purified rGST-JpIocys2a probed with anti-GST-Hl primary antibody and rabbit anti-IgG secondary antibody conjugate with alkaline phosphatase; purified rBrBmcys2b and rBrBmcys2c probed with anti-histidine tag primary antibody conjugate with alkaline phosphatase. Alkaline phosphatase revelations were performed with NBT and BCIP. SDS-PAGE: Recombinant cystatins resolved by 14% SDS-PAGE were stained with Coomassie blue G-250; purified rGST-JpIocys2a before and after thrombin cleavage (rGST and JpIocys2a); purified rJpIocys2a, rBrBmcys2b and rBrBmcys2c. MW: molecular weight.

Soluble recombinant BrBmcys2b, BrBmcys2c, and GST-JpIocys2a were expressed in *E. coli* and purified by affinity chromatography. rGST-JpIocys2a was cleaved from GST-tag by thrombin and the purified rJpIocys2a was recovered (Figure 3). SDS-PAGE showed that the conjugated rGST-JpIocys2a protein weight was about 42 kDa (30 kDa from rGST plus 12 kDa from rJpIocys2a), while the weight of cystatins was approximately 12 kDa. These data are in accordance with *in silico* molecular weights estimation. rBrBmcys2b and rBrBmcys2c were recognized by anti-histidine tag antibodies, and rGST-JpIocys2a was recognized by anti-GST-Hl in Western blot assay. The purified recombinant cystatins were subsequently used in inhibitory and immunization assays.

### rJpIocys2a, rBrBmcys2b, and rBrBmcys2c inhibitory profile

**Figure 4:**
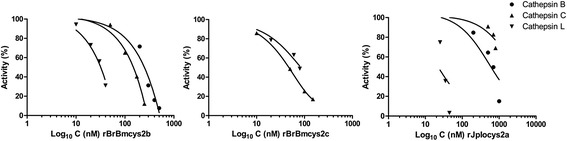
**Activity inhibition assay of cathepsins B, C, and L by rJpIocys2a, rBrBmcys2b, and rBrBmcys2c.** Cathepsins B, C, and L were incubated with Z-Arg-Arg-pNA (0.125 mM), Gly-Phe-pNA (1.8 mM), or Z-Phe-Arg-MCA (0.02 mM), respectively, in the presence of different concentrations of rJpIocys2a, rBrBmcys2b, and rBrBmcys2c. The abscissa shows inhibitors concentration (nM, log_10_); the ordinate shows percentage of remaining enzymatic activity. Incubation of cathepsins B, C, and L without rJpIocys2a, rBrBmcys2b, and rBrBmcys2c represents 100% of enzyme activity.

Inhibitory assays were performed to characterize the specificity of rJpIocys2a, rBrBmcys2b, and rBrBmcys2c for target enzymes (Figure 4 and Table 1). rJpIocys2a, rBrBmcys2b, and rBrBmcys2c modulated the activity of mammal cathepsins B, C, and L at distinct patterns. rJpIocys2a and rBrBmcys2b inhibited all cysteine cathepsins, showing higher affinity for cathepsin L and B, respectively (Table 1). In contrast, rBrBmcys2c did not inhibit cathepsin B, showing higher affinity for cathepsin C. Also, rJpIocys2a, rBrBmcys2b, and rBrBmcys2c inhibited these peptidases with apparent inhibition constants between 0.45 and 154.7 nM. The exception was rJpIocys2a, which shows Ki higher than1 μM for cathepsin C. Cathepsin G, a serine protease, was not inhibited by these recombinant cystatins.

### Recognition of native and recombinant cystatins by hyperimmune sera

**Figure 5:**
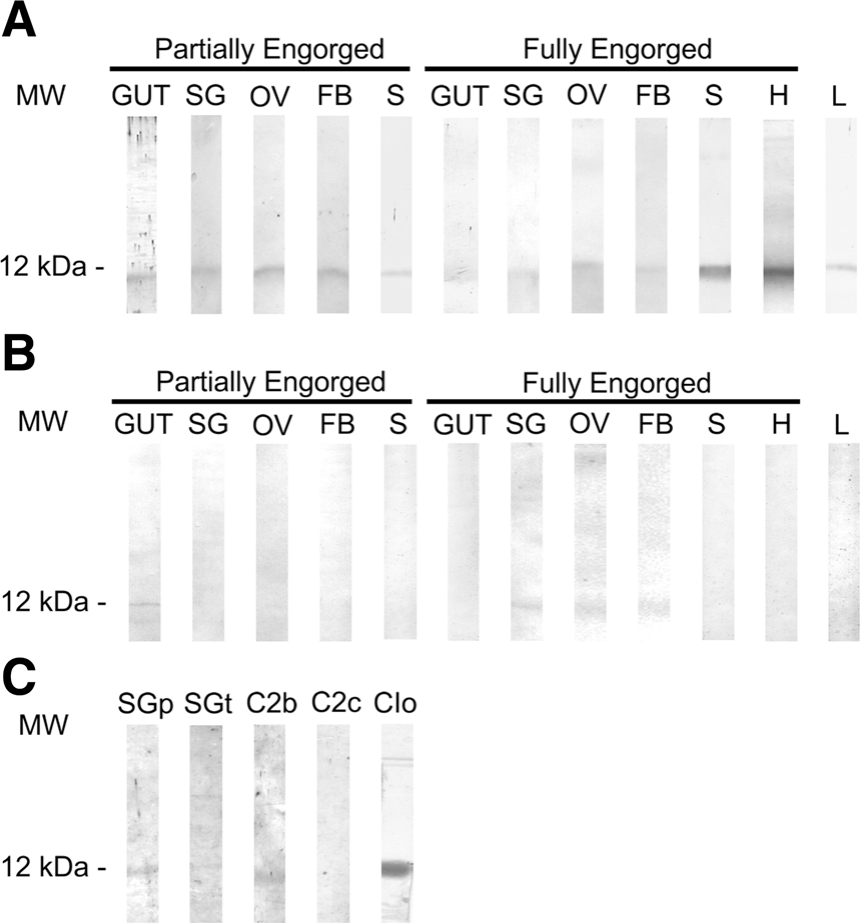
**Cross-immunogenicity between native and recombinant tick cystatins.** By Western blot, *R. microplus* and *I. ovatus* recombinant cystatins or *R. microplus* tissue extracts were analyzed using sera (1:50) against: **A)** rBrBmcys2b; **B)** rBrBmcys2c **C)** STQpep. SG, salivary glands; OV, ovary; FB, fatty body, S, saliva; H, hemolymph; L, larva; SGp, salivary glands from partially engorged female; SGt, salivary glands from fully engorged female; C2b, rBrBmcys2b; C2c, rBrBmcys2c; CIo, rJpIocys2a. MW: molecular weight. Anti-IgG alkaline phosphatase rabbit sera and peroxidase hamster sera conjugates were used as secondary antibodies. Alkaline phosphatase revelations were performed with NBT and BCIP. Peroxidase revelations were performed with DAB, H_2_O_2_ and CoCl_2_.

Sera against STQpep, rBrBmcys2b and rBrBmcys2c were used to determine the presence of cystatins in *R. microplus* tissues as well as the cross-antigenicity between peptide, native, and recombinant cystatins (Figure 5). Native cystatins present in saliva, larvae, ovary, gut, salivary glands, and fat body (apparent molecular mass of 12 kDa) were differentially recognized by these sera. Native cystatins were recognized by anti-rBrBmcys2b in all tissues (Figure 5A), whereas anti-rBrBmcys2c sera recognized cystatins in gut from partially engorged females and in ovary, salivary glands and fat body from fully engorged females (Figure 5B). rBrBmcys2b and native cystatins from partially and fully engorged female salivary glands were recognized by anti-STQpep sera (Figure 5C). The hosts sera inoculated with PBS did not recognize native cystatins in these tissues (data not shown). Furthermore, anti-rBrBmcys2b serum detected cystatin in hemolymph (Figure 5A), unlike anti-rBrBmcys2c and negative controls sera. Since these sera were raised against STQpep and recombinant cystatins, this recognition shows the cross-antigenicity between native and peptide/recombinant cystatins.

### Phylogenetic analysis

**Figure 6:**
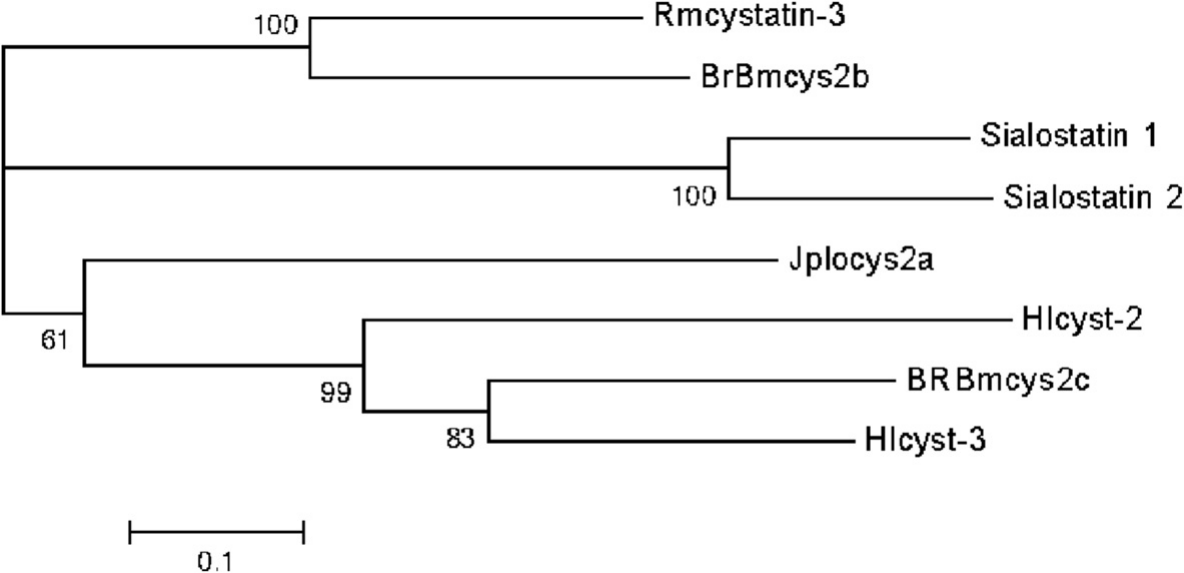
**Phylogenetic analysis of tick cystatins.** *R. microplus* BrBmcys2b [GenBank: KC816580], *R. microplus* BrBmcys2c [GenBank: KC816581], *R. microplus* Rmcystatin-3 [GenBank: AIX97454]; *I. ovatus* JpIocys2a [GenBank: KP253747]; *I. scapularis* sialostatin 1 [GenBank: AF483724], *I. scapularis* sialostatin 2 [GenBank: DQ066048]; *H. longicornis* Hlcyst-2 [GenBank: DQ364159], *H. longicornis* Hlcyst-3 [GenBank: EU426545]. Bootstrap values of 1,000 simulations are shown at the branches.

A neighbor-joining tree constructed with cystatin amino acid sequences from Ixodidae ticks is shown in Figure 6. We selected sequences of cystatins that have inhibitory profile and tissue localization that have been characterized. In the tree constructed, BrBmcys2b and BrBmcys2c grouped with other cystatins present in saliva/salivary glands and gut, respectively, showing tissue localization conservation between cystatin from the two branches of the tree. JpIocys2a grouped with the branch of cystatins expressed mainly in gut, suggesting the importance of this cystatin in tick blood digestion. All these cystatin are cathepsin L inhibitors, whereas cystatins that inhibit cathepsin B and C are present in the two branches.

## Discussion

The function of cystatins in parasite physiological processes is a subject of increasing interest among many research groups [3,49]. In ticks, however, only in recent years did cystatin roles begin to be elucidated [4]. The main goals of these works were the characterization of cathepsin targets, tissue localization and immune system modulation by tick cystatins. To date, no *I. ovatus* and only two *R. microplus* cystatin were biochemically characterized [5,50]. In the present work, we characterized the inhibition profile of one *I. ovatus* and two *R. microplus* cystatins. To uncover the inhibitory profile of these cystatins, we selected three cysteine proteases involved in host hemoglobin proteolytic degradation cascade by ticks [51-53]. In enzymatic inhibition assays, rBrBmcys2b inhibited cathepsins B, C, and L activities, and was detected in all tissues and secretions analyzed, suggesting the broad enzymatic regulation by this inhibitor in *R. microplus* physiology. Among the tissues from partially engorged females analyzed, BrBmcys2c was detected only in gut, suggesting the role of BrBmcys2c in blood metabolism. A potential target for this cystatin could be BmCL1, VTDCE and/or RmLCE, cathepsins from *R. microplus* that were detected in tick gut during feeding stages [19,20,54]. The pattern of native BrBmcys2b and BrBmcys2c expression corroborates previous qPCR results, which showed a higher mRNA transcripts expression in gut, as compared to other tick tissues [22]. The presence of BrBmcys2b in tissue that showed no transcription for this cystatin gene maybe the result of cystatin synthesis in gut, and its subsequent exportation to other tissues, similarly to other tick proteins [21,55,56]. The presence in hemolymph of BrBmcys2b, but not BrBmcys2c, supports this hypothesis. JpIocys2a inhibited cathepsins B, C and L at different levels. Cathepsin L and B were highly inhibited, when compared to C inhibition. The phylogenetic tree analysis show that JpIocys2a grouped together with gut cystatins, indicating that JpIocys2a could be secreted in *I. ovatus* gut lumen. In *Ixodes ricinus* blood digestion, cathepsins B and L hydrolyze hemoglobin secondary large fragments, whereas cathepsin C degrades it down to small fragments [51]. Therefore, JpIocys2a may have major importance during modulation of initial hemoglobin degradation in blood digestion by ticks.

*In vitro* and *in vivo* experiments have demonstrated the role of tick cystatins in the modulation of host immune system components responsible for anti-parasite infestation [10,16,57]. Similarly to Sialostatin L2 [37], rBrBmcys2b and rBrBmcys2c were not recognized by sera of tick-infested hosts (data not shown), indicating that the *R. microplus* cystatins are not immunogenic for bovines when inoculated through tick bite. The presence of BrBmcys2b in *R. microplus* saliva as well as its ability to inhibit cathepsin L suggests the participation of this cystatin in mechanisms to avoid host immune system. Cathepsin L is involved in mammal immune system process by MHC class II-presentation pathway regulation [58], as well as extracellular matrix breakdown during inflammation [59]. Cathepsin L secreted by macrophages inside tick feeding cavity would destroy host tissue elasticity, which is required for the effective parasite attachment [10]. Inhibition of cathepsin L by secreted tick cystatins would help tick feeding, whereas the absence of these compounds would result in inflammation and parasite rejection due to the host’s immune response. This outcome was observed in tick cystatin knock-down and vaccination experiments [8,10,16,37]. Consequently, host cathepsin L inhibition would result in a weaker immune response against tick infestation. The presence of BrBmcys2b in partially and fully engorged female ovary indicated that this cystatin is important during egg development. In ovary, a vitellin degrading cysteine endopeptidase (VTDCE) that plays a crucial physiological role in egg maturation through vitellin mobilization [21,54] is a potential enzyme target for BrBmcys2b.

*In silico* and *in vitro* comparative cross-antigenicity analyses of tick proteins, as CRTs [60], or GSTs [61], showed that, despite their high amino acid sequence conservation between homologues, these proteins display different immunodominant epitopes. Furthermore, antibodies against CRT and GST from *H. longicornis* were able to recognize native and recombinant forms of *R. microplus* homologous proteins [60,61]. Moreover, when GST-Hl was used in a vaccination trial against *R. microplus* infestation in cattle, the number of engorged females decreased by around 50% [61]. To select immunodominant epitopes to explore the potential of cystatin in an anti-tick vaccine, a peptide from JpIocys2a was synthesized based on their antigenicity and similarity with *R. microplus* cystatins, and used for rabbit immunization. This approach seems to be interesting, because the immune response generated against designed peptide could be directed to immunodominant protective epitopes in native proteins. Synthetic peptide based vaccines have been studied as a strategy against malaria [62], hepatitis C [63,64], foot-and-mouth disease [65], human papilloma virus [66] and *Toxoplasma gondii* [67]. Vaccination experiments using synthetic peptide from *R. microplus* Bm86 and *Plasmodium falciparum* also induced antibodies that recognized proteins from which the amino acid sequence had originated [68]. Furthermore, the peptides selected from *R. microplus* Bm86 developed a protection in cattle ranging between 36% and 81% [68]. In this work, STQ-pep immunization generating antibodies that recognize native and recombinant *R. microplus* cystatins showed cross-reactivity in the selected peptide region. The results reveal that the peptide construct is immunogenic, allowing to recognize rJpIocys2a by anti-STQpep antibodies. The cross-reaction of STQ-pep with native cystatins from *R. microplus* salivary glands and rBrBmcys2b paves the way for further works testing this peptide for a cross-antigenic vaccine.

## Conclusions

This work showed the differential presence of cystatins in *R. microplus* tick tissues, and demonstrates that cathepsins B, C, and L are modulated by JpIocys2a, BrBmcys2b, and BrBmcys2c to different degrees. These results suggest distinct tick physiological roles for these cystatins: BrBmcys2b acts during blood meal processing, egg and larva development, and in host immune system modulation; and BrBmcys2c and JpIocys2a act mainly during blood meal processing. Future anti-hemostatic and immunomodulatory experiments will better clarify the importance of JpIocys2a, BrBmcys2b, and BrBmcys2c in tick physiology.
